# Family member incarceration and physical health problems: A longitudinal study among Australian households

**DOI:** 10.1016/j.ssmph.2021.100810

**Published:** 2021-04-29

**Authors:** Steve G.A. van de Weijer, Kirsten L. Besemer, Susan M. Dennison

**Affiliations:** a Netherlands Institute for the Study of Crime and Law Enforcement (NSCR), PO Box 71304, 1008BH Amsterdam, the Netherlands; b School of Criminology and Criminal Justice, Griffith University.Room 3.09, Social Sciences Building (M10), 176 Messines Ridge Road, Mt Gravatt, QLD 4122, Australia; c Griffith Criminology Institute, Griffith UniversityLevel 4 Social Sciences Building (M10) 176 Messines Ridge Road, Mt Gravatt, QLD 4122, Australia

**Keywords:** Incarceration, Imprisonment, Family, Health, Parental imprisonment, Life-course

## Abstract

This study examines the relationship between poor physical health and exposure to family member incarceration. Longitudinal data (2001–2015) from an Australian nationally representative household-based panel study was used (177,312 observations within 26,572 respondents). Hybrid random-effects models showed a strong correlation between poor physical health and family member imprisonment. However, this strong association can be explained for a large part by differences between individuals, since the association of physical health with within-individual changes in family member imprisonment was considerably lower. Nevertheless, the within-individual analyses showed that male sample members were significantly more likely to experience physical health problems in years in which they experienced family member imprisonment, compared to years in which they did not. This association was not found among females. Moreover, no effect of parental imprisonment on the physical health of young sample members was found.

## Highlights

• Imprisonment of a family member is strongly related to poor physical health.
• This association can be explained for a large part by time-constant confounders.
• Males have poorer health in years in which family members are imprisoned.
• Females do not have poorer health in years in which family members are imprisoned.

## 1 Introduction

Over the past two centuries, incarceration rates in most countries have increased (see e.g., Byrne et al., 2015). Consequently, more and more people have a parent, sibling, spouse, or other family member in prison. A growing body of research examines the potential negative consequences of experiencing the imprisonment of a family member, across various life domains. The physical health of these family members, however, is a topic that remains relatively understudied. Understanding the potential for health impacts on families of prisoners is important as poor health may compromise the capacity for family members to care for themselves and any children in the household, impact employment and earning capacity and subsequent financial distress, and place additional burdens and costs on health systems and services. The focus of the current study is on changes in physical health among family members of Australian prisoners.

Glaze and Maruschak (2008) estimated that approximately 1.7 million American children had at least one parent in prison in 2007, amounting to 2.3 percent of all children under the age of 18. In Australia, the country on which the current study focuses, Quilty (2005) estimated that 38,000 children had at least one parent in prison in 2001. Moreover, 145,000 children had ever lost a parent to prison during their lives, representing five percent of all Australian children. As the Australian prison population reached a new peak of 221 prisoners per 100,000 adults in June 2018 (Australian Bureau of Statistics, 2018), the number of children with a parent in prison has likely further increased. Currently, there are no reliable estimates of the number of Australian children and adults who have experienced incarceration of other family members, such as a sibling, son, or daughter. It is probable that such non-parent close family imprisonments affect an even larger group of children and adults.

Almost all literature examining potential negative consequences of experiencing the imprisonment of a family member has focused on children affected by the imprisonment of a parent, while only few studies examined the consequences for other close relatives (see e.g., Besemer et al., 2018; Lee et al., 2014). Previous studies have shown that children with a parent in prison are at an increased risk of behavioral problems (e.g., Geller et al., 2012; Wakefield & Wildeman, 2011), criminal offending (e.g., Murray et al., 2014; Roettger & Swisher, 2011), and of imprisonment in adulthood (Dennison et al., 2017). In addition to these problem behaviors, several previous studies have also shown other poor outcomes among children of imprisoned parents, including mental health problems, drug use, and poor educational performance (e.g., Mears & Siennick, 2016; Murray & Farrington, 2008; Roettger et al., 2011).

It is important to extend the focus of research on the consequences of parental or family incarceration beyond psychological, behavioral, and financial impacts to consider the physical wellbeing of family members. Such research is important for theoretical development as well as identifying appropriate strategies or services that can minimize harmful outcomes arising from parental or family member imprisonment. However, the physical health of family members has received relatively little attention from researchers (Wildeman et al., 2019).

A number of mechanisms have been proposed that could explain a link between imprisonment of family members and poor health outcomes. Turney (2014) used stress process theory to explain the relationship between parental incarceration and childhood health. According to this theory, individuals are differently exposed to social stressors (e.g., incarceration) that negatively impact on their health through disadvantaged social contexts (Pearlin, 1989). Social stressors experienced by family members could also have consequences across generations. For example, Turney (2014) argues that stressors experienced by parents (e.g., incarceration) can have lasting effects on the physical and mental health of their children. Roettger and Boardman (2012) also argue that parental incarceration is a major and long-term stressor for children, which could lead to mental health problems such as anxiety and depression. In line with gender-based theories of internalizing and externalizing behavior (e.g., Leve et al., 2005), they show that a stressor like parental incarceration is related to depression among females but not males. However, they only found this association among non-obese women, and not among obese women. Based on these results, Roettger and Boardman (2012) suggest that some women express internalization through high calorie intake and sedentary lifestyles, as an alternative coping mechanism, resulting in obesity rather than mental health problems.

Another mechanism linking incarceration of a family member to physical health problems is the diminishing financial resources in these families. Wildeman (2012) summarized previous studies that showed parental incarceration decreases the parent's earnings and labor market prospects, increases legal debts, and leads to costs of keeping in touch with the incarcerated parent (e.g., making visits, sending packages). An Australian study found that, because of financial problems, families affected by paternal imprisonment were more likely than other households to have unpaid utility bills, to be unable to afford prescribed medications and dental treatment, and to go without a substantial meal once a day (Besemer & Dennison, 2018). In addition, Dennison and Besemer (2018) showed that Australian children may miss out on sports activities because of financial problems after paternal imprisonment. Similarly, Schwartz-Soicher et al. (2011) found that American families in which the father is incarcerated were more likely to experience material hardship, which included being unable to pay utility bills, not being able to go to a doctor or hospital when needed, and receiving free food or meals. The results from these studies indicate that the financial situation in families of prisoners can lead to living conditions that might be detrimental for one's health.

Although these mechanisms may apply the most to those who experience the incarceration of a parent or a partner, also the imprisonment of other close family members (e.g., siblings, offspring, extended family members) may impact one's health. It is likely that individuals also experience stress when a close relative from outside their own household is sent the prison. Moreover, the costs to keep in touch with imprisoned relatives are not limited to nuclear family members. Other family members may also financially support the household of the incarcerated relative, impacting on their financial resources as well.

In line with these mechanisms, a number of studies in the United States have shown that individuals who experienced imprisonment of their parents were at increased risk to have physical health problems. Using data from the National Longitudinal Study of Adolescent Health (i.e., Add Health) (Lee et al., 2013; Miller & Barnes, 2015; Roettger & Boardman, 2012) and the National Survey of Children's Health (Turney, 2014), these studies found that a history of paternal imprisonment was related to various specific health outcomes in adulthood, including ADD or ADHD; an increased body mass index; bone, joint, or muscle problems; asthma, bronchitis or emphysema; epilepsy or seizure disorder; hearing problems; high blood cholesterol; HIV or AIDS; migraines; posttraumatic stress disorder; obesity; serious injuries; vision problems; and having fair or poor overall health. Moreover, parental incarceration was shown to be related to offspring mortality (e.g., Dowell et al., 2018; Van deWeijer, Smallbone, & Bouwman, 2018; Wildeman, 2012; Wildeman et al., 2014). Although the cause of death was not measured in these studies, the increased mortality rates among those who experience parental incarceration could be an indication of a bad health.

In addition to studies that focus on parental incarceration, some other studies have studied health consequences for those who experience incarceration of any family member. Lee et al. (2014) investigated the cardiovascular health consequences using a sample of American adults. For women, having a family member in prison at the time of the survey was related to obesity, heart attacks or strokes, having a fair or poor health, diabetes, and hypertension, although the relationships with the latter two did not remain significant after adjusting for possible confounders. However, they did not find any significant associations among the men in their sample. In contrast, White et al. (2016) did find an association between family member incarceration during childhood and having a heart attack in adulthood among American men, but not among the women in their study. Finally, Gjelsvik et al. (2014) found a positive relationship between childhood exposure to the incarceration of a household member and adult self-reported health-related quality of life, among American adults.

However, these previous studies are limited in two important ways. First, it remains unknown whether these associations in previous studies also reflect causal effects. Some of the previously found associations were no longer significant after controlling for confounding variables (Miller & Barnes, 2015; Turney, 2014), suggesting a spurious relationship rather than a causal effect. Establishing causality is further impeded because in almost all previous studies the health of respondents was only measured after the incarceration of their family members (for an exception see Roettger & Boardman, 2012). As a consequence, it is impossible to test whether imprisonment of family members leads to a decrease in physical health or whether poor physical health was present prior to family imprisonment. In addition, almost all previous studies measured health in adulthood and family member imprisonment in childhood (for an exception see Lee et al., 2014) and, thus, focused on long term health outcomes rather than on the immediate consequences of family member imprisonment.

Second, our current knowledge on the relationship between the imprisonment of family members and physical health is further limited by the fact that all studies on this specific topic were conducted in the United States^1^. Differences between the United States and other western countries, in terms of incarceration rates, prison conditions, social security expenditures, and health care systems, might limit the generalizability of the results of previous studies to other western countries. For example, in Australia, the public health insurance scheme (Medicare), provides for fee-free treatment for a public patient in a public hospital by a hospital-appointed doctor. Outside of hospitals, Medicare provides free access (or 100% reimbursement) to general practitioners and 85% reimbursement for specialist services. In addition, the Australian Government subsidizes a wide range of prescription medications under the Pharmaceuticals Benefit Scheme (Australian Institute of Health and Welfare, 2016). This national public health scheme, as well as the existence of a standard minimum wage and comprehensive welfare support, might mean that families affected by incarceration are buffered from potentially deleterious health consequences of reduced household income.

The current study addresses these limitations by using a large, longitudinal (2001–2016) household panel from Australia to examine the relationship between the incarceration of family members and physical health. This study design enables us to examine whether within-individual changes in exposure to the imprisonment of family members over time are related to immediate within-individual changes in physical health. By focusing on within-individual changes over time we control for all, measured and unmeasured, time-stable confounders. Our results will therefore not be affected by pre-existing differences between individuals and will give a better estimate of the actual effect of family member imprisonment on physical health problems. The importance of using such an analytic approach is illustrated by a recent study of Besemer et al. (2018), who found that significant associations of family member imprisonment with mental health problems and social exclusion disappeared after controlling for unmeasured confounders.

Moreover, the current study will also examine whether the associations between poor physical health outcomes and the imprisonment of family members are different for men and women, as some previous studies found such gender differences (e.g., Lee et al., 2014; Roettger & Boardman, 2012; White et al., 2016). In sum, the research questions are twofold: (1) To what extent is exposure to the incarceration of a family member associated with poor physical health? (2) Are these associations the same for men and women?

## 2 Methods

### 2.1 Sample

In order to answer these research questions, data from the Housing, Income, and Labour Dynamics in Australia (HILDA) Survey was used. The HILDA Survey started in 2001 with a large national probability sample of Australian households and contains longitudinal data on a wide range of aspects of life around family dynamics, economic and subjective well-being, and labour market dynamics. Data are collected about each household member, and household members aged 15 years and over are annually interviewed, usually in person. The sample in wave 1 consisted of 19,914 individuals, who formed the basis of the panel to be pursued in each subsequent wave. The sample was extended by including new household members resulting from changes in the composition of the original households and by adding 2153 new households in wave 11. For more information about the HILDA Survey see Summerfield et al. (2016).

Respondents were also asked to fill in a self-completion questionnaire (SCQ) containing more sensitive questions, including questions about physical health, the dependent variable in our analyses. Questions about the imprisonment of family members were not asked in the SCQ of the first wave. Therefore, only data from wave 2–15 (collected between 2002 and 2016) were used in the current study. Across these waves, the SCQ was completed 181,865 times. The cases with missing values on physical health (N = 2549; 1.4%), family member imprisonment (N = 2356; 1.3%), and marital status (N = 6; 0.0%) were excluded from the analyses. This resulted in an analytic sample of 26,572 respondents who were surveyed during a total of 177,312 survey years, an average of 6.7 waves per person.

### 2.2 Measurements

#### 2.2.1 Dependent variable

The Short Form-36 (SF-36) physical functioning scale was used to measure physical health problems. This scale is a subscale from the SF-36 general health measure and was measured in all waves. This scale has been shown to be the best all-around measure of physical health (Ware, 2000), and has an excellent internal consistency reliability (Cronbach's alpha = .92) in our sample. The physical functioning scale is based on ten items about activities that respondents might do during a typical day, such as ‘Walking more than 1 km’ and ‘Lifting or carrying groceries’. Respondents could indicate whether their health limits them now in these activities by answering ‘Yes, limited a lot’, ‘Yes, limited a little’, and ‘No, not limited at all’. Following the SF-36 scoring instructions, the score on the physical functioning scale is then determined by scoring 0 points for each answer in the first category, 50 points for each answer in the second category, and 100 points for each answer in the third category. This leads to an average score on this scale between 0 and 100, with a low score indicating more limitation in physical functioning and a high score indicating less limitation in physical functioning. Since the distribution of the physical functioning scale was heavily left skewed in the HILDA sample (i.e., many respondents having little or no limitation in physical functioning) this dependent variable was dichotomized, with the lowest quartile of scores (which equals all mean scores between 0 and 75) within the sample considered as limited physical functioning. Moreover, three additional dependent variables were constructed based on other dichotomizations, in order to examine the robustness of the results. These additional dependent variables define the lowest 10 (all mean scores between 0 and 45), 30 (all mean scores between 0 and 80), and 50 per cent of scores (all mean scores between 0 and 90) as limited physical functioning.

#### 2.2.2 Independent variables

Two different independent variables were used in the analyses: *close family member imprisonment* and *parental imprisonment*. In the self-completion questionnaire, respondents were asked to indicate whether they had experienced a number of events in the past year, including being detained in a jail/correctional facility and having a close family member detained in a jail/correctional facility. If a respondent indicated that a close family member had been detained in a jail/correctional facility in the past year he or she scored 1 on the independent variable *close family member imprisonment*, while they scored 0 when they did not experience this. Who these close family members are is not defined in the questionnaire and it, thus, includes everyone that the respondent considered as a close family member. However, due to the study design of HILDA study, it is possible to identify cases of parental imprisonment. If a parent in a household indicated that he or she had been detained in a jail/correctional facility in the past year, the children in that household (the respondents) scored 1 on *parental imprisonment* in that particular year. Only respondents up to the age of 21 got a valid score on the independent variable *parental imprisonment* since information on parents was largely unavailable for older respondents.

#### 2.2.3 Control variables

In addition to these independent variables, several control variables, that might be related to both incarceration risk and physical health, were included in the analyses. *Age* was measured as the age on June 30th in the year the survey wave commenced. The variable *Children in household* indicates how many persons between 0 and 17 years old were living in the household in each survey year. Where the respondent was between 15 and 17 years old, this number included the respondent. *Household income* was measured as the OECD-equivalised income, indicated by the percentage difference from the median income in each wave. Households without an income scored −100, households with an income equal to the median income scored 0, and households with an income higher than the median received a positive score. In order to control for outliers, a maximum score of 1000 was used for all households that earned ten times the median income or more. *Marital status* is a categorical variable comprising four categories (i.e., in a relationship, separated, widowed, and single), indicating the marital status at the moment of data collection in each wave. *Employment status* consisted of three categories (i.e., employed, unemployed, and not in the labour force) indicating whether respondents were employed or not in each wave. Finally, the variable *male* was included as a time-constant control variable, indicating whether a respondent was male or female. This variable was used to split up analyses between male and female respondents.

### 2.3 Analyses

In order to assess the relationship between poor physical health and the imprisonment of parents and family members, we constructed a person-year file, recording separate information for each year a respondent was surveyed. This person-year file was used to estimate hybrid random-effects models (also called Between-Within models), which combine the advantages of both random and fixed effects models (Bell & Jones, 2015). This hybrid model can be written as (Schunck, 2013):

$$y_{it}=\beta_{0}+\beta_{1}\left(x_{it}-{\bar{x}}_{i}\right)+\beta_{2}c_{i}+\beta_{3}{\bar{x}}_{i}+\mu_{i}+\varepsilon_{it}$$

where subscript *i* denotes individual respondents and subscript *t* denotes the years in which a sample member was observed. *β*_*1*_ gives the within-individual effect estimates of time-varying variables *x*_*it*_, which are equal to the estimates that would have been found when a fixed effects model was used. This within-individual effect is estimated by transforming scores on time-varying variables into deviations from respondents' person-specific means (i.e., $\bar{x}$_*i*_). By subtracting the person-specific means, only within-person changes are regressed. The model therefore takes into account structural differences between individuals' health (e.g., due to chronic diseases or old injuries) and automatically controls for bias caused by all observed and unobserved time-invariant variables (i.e., all time-stable differences between respondents). For the parental imprisonment variable, for example, the exponential *β*_*1*_ indicates the increase or decrease in the odds to have a limited physical functioning in a year someone experienced parental imprisonment compared to a year in which this same person did not experience parental imprisonment. In a regular fixed effect model, it is not possible to include variables that are constant over time and, thus, do not change within individuals. In the hybrid random-effects models, however, estimates of such time-invariant variables can be included, and these are reflected by *β*_*2*_ in the formula. It is important to note that the *β*_*2*_ estimates are not automatically controlled for unobserved time-invariant variables. *β*_*3*_ gives the between-individuals effect estimates of time-varying variables *x*_*it*_, and indicates the association between the person-specific means (i.e., $\bar{x}$_*i*_) and a limited physical functioning (i.e., *y*). For the parental imprisonment variable, for example, the exponential *β*_*3*_ indicates the increase or decrease in the odds to have a limited physical functioning for a respondent who experienced parental imprisonment during all years (i.e., $\bar{x}$_*i*_ = 1) and a respondent who did not experience this during any wave (i.e., $\bar{x}$_*i*_ = 0). Similar to the *β*_*2*_ estimates, the *β*_*3*_ estimates are not automatically controlled for unobserved time-invariant variables.

Analyses were first conducted on the total sample, with all respondents with a valid score on the dependent and independent variables. Next, analyses were repeated among respondents between the age of 15 and 21, since a stressful event like the imprisonment of family members, and parental imprisonment in particular, might have a stronger impact on young respondents. Moreover, analyses were conducted separately for males and females to examine whether there are gender differences in the association between the imprisonment of family members and physical health. In addition to the odds ratios from the hybrid models, also marginal effects were estimated which indicate the probability of having a limited physical functioning for those with and without an imprisoned family member.

Finally, all analyses were repeated using three other dependent variables based on different dichotomizations of the physical health scale, in order to examine the robustness of the results. In addition to the dichotomization of the main analyses in which the lowest 25 per cent of scores was considered as limited physical functioning, those with the lowest 10, 30 and 50 per cent of scores were defined as limited physical functioning in these robustness analyses. All analyses were conducted in Stata version 15.

## 3 Results

Table 1 shows the descriptive statistics of all variables, across all waves, that were used in the analyses. A quarter of respondents were limited in their physical functioning.^3^ In relatively few years the respondents experienced the imprisonment of a close family member (1.4%) or a parent (0.2%). The age of sample members varied between 15 and 101, with an average age of 44.38 years. Moreover, the respondents on average had 0.77 children living in their household and the average household income was 22 per cent higher than the median household income. Furthermore, most respondents were either in a relationship or single and were either employed or not in the labour force. Just less than half of the respondents were male.

**Table 1:** Descriptive statistics of all variables used in hybrid random-effects regression models.

Dependent variable	Mean	S.D.	Minimum	Maximum	N
*Limited physical functioning*	25.3%		0	1	177,312
**Independent variables**					
*Close family member imprisonment*	1.4%		0	1	177,312
*Parental imprisonment*	0.2%		0	1	23,938
**Control variables**					
*Age*	44.38	18.34	15	101	177,312
*Children in household*	0.77	1.13	0	12	177,312
*Household income*	22.82	90.57	−100	1000	177,312
*Marital status*					177,312
In a relationship	63.4%		0	1	
Separated	8.7%		0	1	
Widowed	4.7%		0	1	
Single	23.2%		0	1	
*Employment status*					177,312
Employed	64.1%		0	1	
Unemployed	3.6%		0	1	
Not in the labour force	32.3%		0	1	
*Male*	46.8%		0	1	177,312

Table 2 shows the results of the logistic hybrid random-effects regression models in which limited physical functioning was predicted. Model 1 shows that, among all sample members, both between-individual differences (Odds Ratio = 5.26) and within-individual changes (OR = 1.16) in family member imprisonment are associated with increased odds to have a limited physical functioning. The odds ratio for between-individual differences indicates that respondents who experience family member imprisonment in every wave (i.e., mean score is 1) have 5.26 times the odds to be limited in their physical functioning than respondents who never experience family member imprisonment (i.e., mean score is 0). The estimated marginal effects show that those who have family members imprisoned in every wave have a probability to have a limited physical functioning of 42.6 per cent, while this probability is only 23.4 per cent for those who never experienced the imprisonment of a family member.

**Table 2:** Logistic hybrid random-effects regression models on limited physical functioning.

Variables	Model 1: All respondents		Model 2: Males		Model 3: Females	
**Within-individual change**	OR	SE^a^	OR	SE	OR	SE
Family member imprisonment	1.16*	0.09	1.45**	0.19	1.03	0.10
Age	1.09***	0.00	1.08***	0.00	1.10***	0.00
Children in household	0.92***	0.01	1.03	0.02	0.84***	0.02
Household income	0.99**	0.00	0.99**	0.00	1.00	0.00
Marital status						
In a relationship	(ref.)		(ref.)		(ref.)	
Separated	1.05	0.07	1.20*	0.12	1.01	0.08
Widowed	1.50***	0.14	2.08***	0.38	1.31**	0.14
Single	1.16**	0.07	1.40***	0.13	1.01	0.08
Employment status						
Employed	(ref.)		(ref.)		(ref.)	
Unemployed	1.25***	0.07	1.31***	0.11	1.21**	0.09
Not in the labour force	1.74***	0.06	2.21***	0.12	1.51***	0.06
**Between-individual differences**						
Family member imprisonment	5.26***	1.13	6.71***	2.30	4.60***	1.27
Age	1.06***	0.00	1.06***	0.00	1.07***	0.00
Children in household	0.90***	0.02	0.98	0.03	0.84	0.03
Household income	0.99***	0.00	0.99***	0.00	0.99***	0.00
Marital status						
In a relationship	(ref.)		(ref.)		(ref.)	
Separated	1.72***	0.15	1.69***	0.23	1.73***	0.19
Widowed	1.96***	0.23	0.97	0.24	2.19***	0.30
Single	1.09	0.07	1.14	0.11	0.98	0.09
Employment status						
Employed	(ref.)		(ref.)		(ref.)	
Unemployed	5.91***	0.86	4.30***	0.84	8.24***	1.78
Not in the labour force	9.73***	0.62	12.11***	1.17	8.49***	0.73
Male	0.88**	0.04				
Intraclass correlation	0.67		0.66		0.67	
McKelvey & Zavoina Pseudo R^2^	0.30		0.28		0.31	
N (individuals)	26,572		12,790		13,782	
N (observations)	177,312		82,917		94,395	

*Note:* *p < .05; **p < .01; ***p < .001 (one-sided).

a Estimates of the standard errors of the odds ratios were derived in Stata, using the delta rule. The standard errors by delta rule is: se(OR_b_) = exp(b) * se(b).

The odds ratio for within-individual change indicates that respondents who experience imprisonment of a close family member have 1.16 times the odds to be limited in their physical functioning in the years that they experience family member imprisonment, compared to the years that they do not. Marginal effects show that, for a respondent who experienced family member imprisonment in 10 per cent of the waves, the probability to have a limited physical functioning is 26.5 per cent in years in which a family member was imprisoned, and 24.9 per cent when this was not the case. Although the odds ratio for within-individual changes is also significant it is considerably and significantly (p < .001) smaller than the odds ratios for between-individual changes. This indicates that a large part of the overall association between limited physical function and the imprisonment of close family members can be explained by differences between individuals. The intraclass correlation also indicates that about two third of the variance in limited physical functioning can be attributed to differences between individuals.

Within-individual changes in all control variables are also significantly related to limited physical functioning. Aging is associated with increased odds to be limited in physical functioning. An increase in the number of children in the household and an increase in the household income, on the other hand, are both negatively related to a limited physical functioning. Moreover, respondents who become a widow or single instead of married, and who become unemployed or are otherwise not in the labour force, are all at increased risk to have limited physical functioning. Finally, males are less likely to have a limited physical functioning compared to females.

Next, the regression models on limited physical functioning are estimated separately for males (Model 2) and females (Model 3). Model 2 shows that both between-individual differences (OR = 6.71) and within-individual changes (OR = 1.45) in family member imprisonment are positively associated with a limited physical functioning among male sample members. Thus, males have a 45 percent higher odds to have a limited physical functioning in the years that they experience the imprisonment of a close family member, compared to the years that they do not experience this. The results in Model 3 show that, among female sample members, only the between-individual differences in family member imprisonment (OR = 4.60) are significantly related to a limited physical functioning. The insignificant odds ratio for within-individual changes in family member imprisonment indicates that women, in contrast to men, are not significantly more likely to have a limited physical functioning in the years that they experience the imprisonment of a close family member. These regression coefficients for within-individual change for men and women are significantly different from each other (p < .05).

In additional analyses, the hybrid models from Table 2 were estimated again using dependent variables with different dichotomizations of the physical health scale. Overall, the results of these analyses (not shown in Table 2) show that the odds ratios for within-individual changes in family member imprisonment increase when less strict dependent variables are used (i.e., when more respondents are defined as having limited physical functioning). The conclusions with respect to both family member imprisonment variables do not change, with two exceptions. When only the lowest decile of scores are considered as limited physical functioning, the odds ratio for within-individual change in family member imprisonment is not significant anymore among all respondents (Model 1). Moreover, when the lowest half of scores are defined as limited physical functioning, the odds ratio for within-individual change in family member imprisonment is significant for women (OR: 1.21, p < .05).

Since the imprisonment of parents in particular might have a big impact on the lives of their offspring, the association between parental imprisonment and limited physical functioning is examined in Table 3. In these analyses, only respondents between the age of 15 and 21 who lived in the same household as their parents were included. Model 1 shows that between-individual differences in parental imprisonment (OR = 21.63) are positively related to limited physical functioning. This indicates that youngsters who ever experienced parental imprisonment are more likely to have a limited physical functioning compared to those who never experienced this: the probability to have a limited physical functioning is 47.7 per cent for those who experienced parental imprisonment in all waves, while this probability is only 11.3 per cent for those who never experienced parental imprisonment.

**Table 3:** Logistic hybrid random-effects regression models on limited physical functioning, among young sample members (15–21 years).

Variables	Model 1: All respondents		Model 2: Males		Model 3: Females	
**Within-individual change**	OR	SE	OR	SE	OR	SE
Parental imprisonment	2.49	1.41	1.85	1.31	3.93	3.75
Age	0.88***	0.02	0.90**	0.03	0.86***	0.03
Children in household	0.94	0.04	0.95	0.06	0.93	0.06
Household income	1.00	0.00	1.00	0.00	0.99*	0.00
Marital status						
In a relationship	(ref.)		(ref.)		(ref.)	
Single	0.80	0.11	0.99	0.25	0.71*	0.12
Employment status						
Employed	(ref.)		(ref.)		(ref.)	
Unemployed	0.92	0.10	0.91	0.14	0.92	0.14
Not in the labour force	1.11	0.10	1.02	0.16	1.03	0.12
**Between-individual differences**						
Parental imprisonment	21.63**	27.11	34.96**	51.90	9.62	22.29
Age	1.03	0.03	1.02	0.05	1.02	0.05
Children in household	1.23***	0.04	1.21***	0.06	1.22***	0.07
Household income	0.99***	0.00	0.99***	0.00	0.99***	0.00
Marital status						
In a relationship	(ref.)		(ref.)		(ref.)	
Single	0.40***	0.05	0.61**	0.13	0.33***	0.06
Employment status						
Employed	(ref.)		(ref.)		(ref.)	
Unemployed	3.24***	0.53	2.32***	0.52	4.41***	1.06
Not in the labour force	2.36***	0.28	1.38*	0.23	3.58***	0.59
Male	1.03	0.08				
Intraclass correlation	0.55		0.53		0.56	
McKelvey & Zavoina Pseudo R^2^	0.05		0.03		0.09	
N (individuals)	7220		3536		3684	
N (observations)	23,938		11,486		12,451	

*Note:* *p < .05; **p < .01; ***p < .001 (one-sided).

The odds ratio for within-individual change, however, is considerably smaller and not significant, which indicates that the association between limited physical function and parental imprisonment can be explained by differences between individuals rather than by changes within individuals over time. Models 2 and 3 show that also in the separate analyses for males and females no significant results were found for within-individual changes in parental imprisonment. However, the effect sizes for parental imprisonment as shown in Table 3 are considerably larger than the effect sizes for family member imprisonment in Table 2. The lack of significant results for parental imprisonment might therefore be the consequence of the decreased statistical power after selecting a sub-sample and focusing on a less prevalent outcome variable.

The hybrid models from Table 3 were also estimated again using dependent variables with different dichotomizations of the physical health scale. The conclusions with respect to both parental imprisonment variables do not change, except when the lowest half of scores are defined as limited physical functioning. Using this dependent variable, the odds ratio for within-individual change in parental imprisonment is significant for women (OR: 8.78, p < .05).

## 4 Discussion

In this paper we examined the relationship between experiences of the imprisonment of family members and poor physical health. Moreover, we explored whether these associations differed between males and females. By using longitudinal panel data and hybrid random-effects models, we were able to distinguish between associations due to between-individual differences and associations due to within-individual change. These latter associations give a better estimate of the true causal effect of family member imprisonment on physical health, since they control for all time-stable confounders.

First, the relationship between the imprisonment of close family members and limited physical functioning of respondents was studied. The results show that between-individual differences between the imprisonment of close family members were strongly related to an increased risk of having limited physical functioning. However, the odds ratio for the within-individual changes was considerably lower, which indicates that the strong association between limited physical functioning and the imprisonment of relatives is mainly due to differences between individuals. As the within-individual comparison controls for time-stable confounders, the low odds ratio suggests that the association is for a large part spurious rather than causal. These results emphasize the importance of using study designs that also control for unmeasured bias. As many of the previous studies on this topic use cross-sectional designs that are not able to control for such bias, one could expect the associations that were found in these studies to be smaller when such bias is filtered out. Nevertheless, even after controlling for all time-stable bias, the within-individual effects remained significant and indicates that Australians who experience the imprisonment of a close family member have worse physical functioning in the years that this imprisonment occurs compared to years in which it did not occur.

One possible mechanism behind this relationship between incarceration of family members and poor physical functioning could be financial problems in these families, since previous studies have showed that reduced household income and increased expenditure in families of prisoners can lead to living conditions that might be detrimental to health (Besemer & Dennison, 2018; Dennison & Besemer, 2018; Schwartz-Soicher et al., 2011). Although it was beyond the scope of the current study to examine the mechanisms behind this relationship, household income was included in the analyses as a control variable. As expected, a decrease in the household income was related to an increase in physical functioning problems. Nevertheless, significant effects of close family member imprisonment on limited physical functioning were found, even after controlling for the household income. A decreased income because of the imprisonment could, thus, not explain these relationships.

Gender specific analyses showed that the within-individual effects of the imprisonment of a close family member were more often found among male respondents, and were also significantly stronger than among female respondents. In a previous study using the same dataset, Besemer et al. (2018) also found that males were more likely to have mental health problems after the imprisonment of a close family member, while females were not. The results from these Australian studies are not in line with American studies, which more often find poor health outcomes among females who experience the imprisonment of family members (e.g., Lee et al., 2014; Roettger & Boardman, 2012). Future studies should investigate why family member imprisonment in Australia is stronger associated with poor health outcomes among males than females.

Finally, no significant within-individual effects of parental imprisonment were found on limited physical functioning, despite a strong between-individual association. The associations with parental imprisonment were stronger than those with close family member imprisonment, which suggests that the lack of significant results in the within-individual analyses could be the consequence of the relatively low prevalence of parental imprisonment in this sample from the general population. Since only individuals who have varying scores on parental imprisonment (i.e., experienced parental imprisonment in at least one year) contribute to the within-individual regression coefficient, the statistical power of these analyses is limited. Therefore, samples with a higher prevalence of imprisoned parents and family members are necessary to test whether the imprisonment of parents has a stronger association with poor health outcomes than the imprisonment of other family members.

### 4.1 Strengths and limitations

This study adds to the literature in several ways. First, all previous studies on the relationship between the imprisonment of relatives and poor physical health were all conducted in the United States (e.g., Lee et al., 2013; Miller & Barnes, 2015; Roettger & Boardman, 2012; Turney, 2014). Given the differences in incarceration rates, prison conditions, social security expenditures, and health care systems between United States and other countries, it is necessary to test whether results from these studies are replicated in other countries. By using an Australian sample, this is the first study on this topic based on a non-US sample. Second, in almost all previous studies the physical health of respondents is only measured after the incarceration of their family members (for an exception see Roettger & Boardman, 2012). By using panel data, with measurements in up to 14 waves, and hybrid random-effects models, this study was able to test whether imprisonment of family members is related to a change in physical health, thereby controlling for selection effects where individuals may have already had poor physical health prior to the imprisonment of their relatives. Third, this study focused on the immediate health consequences of the imprisonment of a family member, while almost all previous studies focused on long term consequences as they measured the association between childhood family member imprisonment and adulthood health problems.

Besides these strengths, the current study is also limited in some ways. First, only imprisonment of family members was measured in this study, while information on criminal behavior of family members was unavailable. Since criminal behavior logically precedes a prison sentence, the results in this study could also reflect a relationship between physical health and criminal behavior of family members, rather than between physical health and family member imprisonment. This same problem occurs in almost all previous studies on this topic, as data on the criminal behavior of family members is also missing in these datasets. For future studies it is therefore desirable if poor health outcomes are compared between persons with imprisoned family members, with criminal but non-imprisoned family members, and with non-criminal family members. Moreover, future studies should take into account differences within the group of persons with imprisoned family members, with respect to incarceration length. It can be expected that a short prison sentence has less detrimental effects than long sentences. In the current study this could not be tested since information on the length of the imprisonment of family members was unfortunately unavailable.

Second, although this study used a hybrid random-effects model in order to distinguish between-individual differences and within-individual changes, the results cannot be interpreted as causal effects. The fixed effects in these models (i.e., the within-individual changes) control for all bias caused by time-constant confounders, but not for time-varying confounders. In the analyses, some potential time-varying confounders have been included as control variables (e.g., employment status, marital status, age, income), but it is impossible to control for unmeasured time-varying confounders (e.g., illicit drug use). It is therefore possible that some of the significant results in this study are the consequent of such unmeasured time-varying confounders and therefore do not reflect causal effects.

Third, this study focuses on the short-term consequences of the imprisonment of family members. The hybrid random-effects model shows whether a change in the imprisonment of relatives in a certain year is related to changes in physical health in the same year. However, a stressful life event such as the imprisonment of a family member could also possibly have consequences over the long term. Such long-term effects are not examined in the current study; datasets with a much longer follow-up period are required to capture such effects. Moreover, it could be expected that those who are repeatedly exposed to incarcerations of family members might be more likely to experience health problems. It was beyond the scope of the current study to examine the effects of repeat incarcerations, but this is an interesting and important topic for future studies.

Finally, even though a very large sample (N = 26,572) from the general Australian population was used, the number of respondents who reported that they had been imprisoned or experienced the imprisonment of a close family member was relatively small. Consequently, the statistical power of our analyses might have been too small to identify smaller effects of the imprisonment of parents and close family members on poor health outcomes. In addition, the underreporting of imprisonment might bias the results. If respondents do not report their own or their family members' imprisonment this could lead to an underestimation of the true effect of the imprisonment of family members on respondents’ physical health. Moreover, also the number of respondents who reported to be Indigenous Australians was very low, which made it impossible to examine differential effects by Indigenous status.

## 5 Conclusion

In conclusion, the results from this study show that the imprisonment of parents and family members is associated with health outcomes, but that these associations could, for a large part, be explained by differences between respondents and therefore do not necessarily reflect causal effects. This illustrates the importance of using quasi-experimental research designs that control for hidden bias, such as the fixed effects analyses in the current study, when examining the consequences of the imprisonment of family member. Some of these results were inconsistent with the existing literature, which is mostly based on American studies, and therefore shows that results from the United States are not always generalizable to other countries, particularly those with lower incarceration rates, stronger public health care systems, and higher social security expenditures. Further cross-national comparisons are required to determine whether our findings are replicable in other countries.

Nonetheless, the results of the current study add to a vast and growing amount of studies which show that the experience of imprisonment of family members is related to various poor outcomes (e.g., Besemer et al., 2018; Mears & Siennick, 2016; Murray & Farrington, 2008; Roettger et al., 2011). This vulnerable group requires greater support and help to limit these poor outcomes. However, since our within-individual analyses showed that the link between family member imprisonment and poor physical health is largely spurious, potential interventions aimed specifically at the imprisonment will probably not have an immediate positive impact on the physical health of family members of prisoners.

## Declarations of interest

None.

## Author statement

Steve van de Weijer: Conceptualization, Formal analysis, Writing - original draft.

Kirsten Besemer: Conceptualization, Formal analysis, Writing – Review & editing.

Susan Dennison: Conceptualization, Writing – Review & editing, Funding acquisition.

## Footnotes

1 Previous studies from outside the United States have examined mortality as outcome, but did not specifically look at physical health problems.
